# Supplementary material for: Aerosol–Cell Exposure System Applied to Semi-Adherent Cells for Aerosolization of Lung Surfactant and Nanoparticles Followed by High Quality RNA Extraction
Source: Nanomaterials (Basel). 2022 Apr 15;12(8):1362. doi: 10.3390/nano12081362 (PMC9028274; doi:10.3390/nano12081362)
Supplement: Supplementary file 1 [file nanomaterials-12-01362-s001.zip › nanomaterials-1605611-supplementary.pdf]

Supplementary Data

Aerosol–Cell Exposure System Applied to Semi-Adherent Cells for Aerosolization of Lung Surfactant and Nanoparticles Followed by High Quality RNA Extraction

Mélanie M. Leroux[1,*,†], Romain Hocquel [1], Kevin Bourge [1], Boštjan Kokot [2], Hana Kokot [2], Tilen Kokliˇc [2], Janez Štrancar [2], Yaobo Ding [3], Pramod Kumar [3], Otmar Schmid [3], Bertrand H. Rihn [1], Luc Ferrari [1] and Olivier Joubert [1]

1 Université de Lorraine, CNRS, IJL, F-54000 Nancy, France ; romain.hocquel@univ-lorraine.fr (R.H.); kevin.bourge@univ-lorraine.fr (K.B.); bertrand.rihn@univ-lorraine.fr (B.H.R.); luc.ferrari@univ-lorraine.fr (L.F.); olivier.joubert@univ-lorraine.fr (O.J.)

2 Jožef Stefan Institute, Department of Condensed Matter Physics, 1000 Ljubljana, Slovenia; bostjan.kokot@ijs.si (B.K.); hana.kokot@ijs.si (H.K.); tilen.koklic@ijs.si (T.K.); janez.strancar@ijs.si (J.Š)

3 Institute of Lung Health and Immunity, Helmholtz Zentrum München, German Research Center for Environmental Health, 85764 Neuherberg, Germany; dyb450303@gmail.com (Y.D.); pramod.kumar@helmholtz-muenchen.de (P.K.); otmar.schmid@helmholtz-muenchen.de (O.S.)

***** Correspondence: melanie.leroux.pro@gmail.com and melanie.lovera-leroux@univ-lorraine.fr

† Institut Jean Lamour, UMR CNRS 7198, 2 Allée André Guinier BP 50840, 54011 Nancy, France.

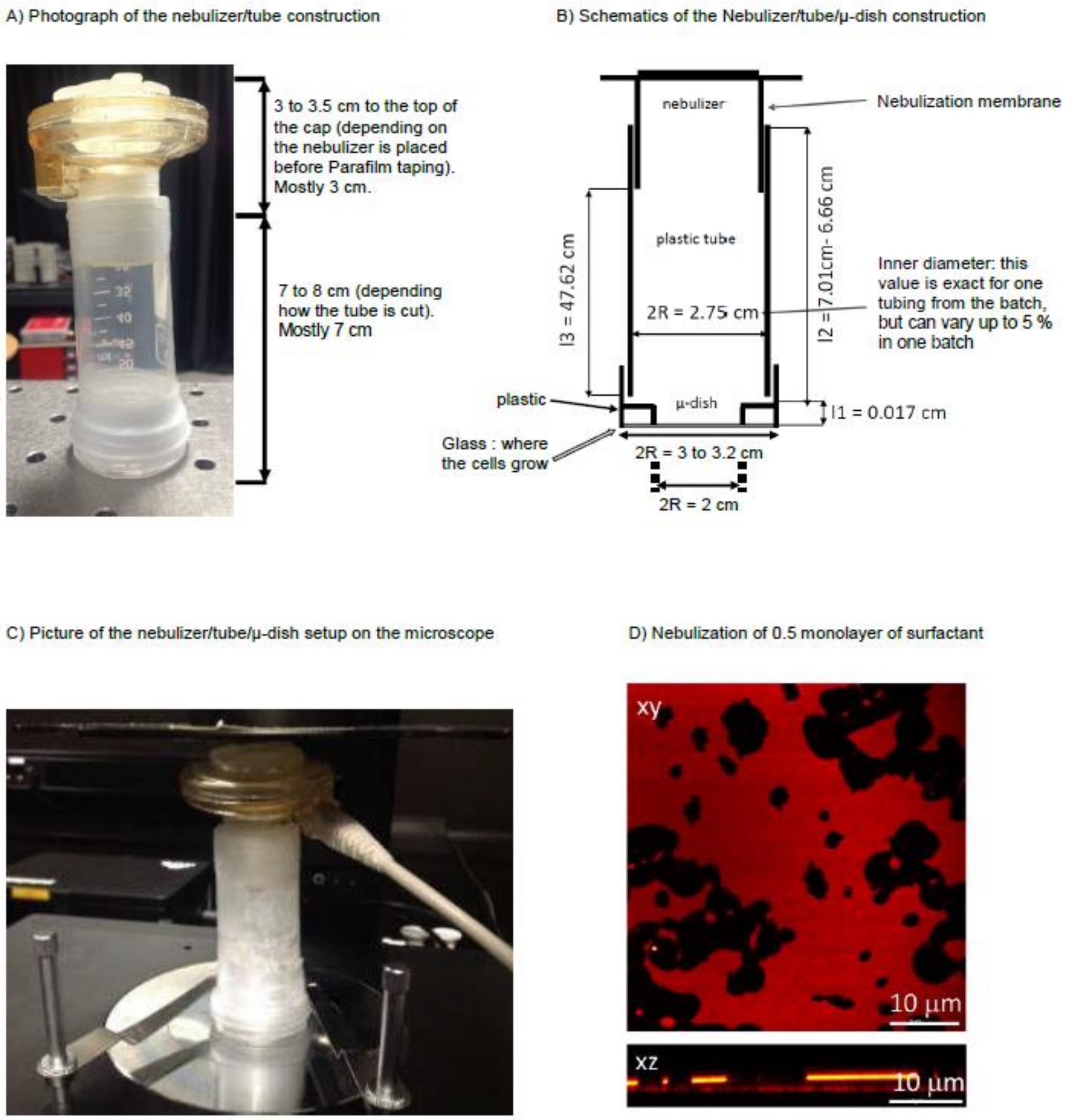

Figure S1: Nebulizer/tube/µ-dish setup for microscopy.

The estimation of the fraction of nebulized surfactant which reaches the sample was performed by nebulizing 12 µg of surfactant (100 µL of 0.12 µg/mL surfactant), resulting in approximately 0.5 monolayers of surfactant on the sample (red area on Supp Data 1D). Considering that a lipid monolayer has a surface area of 500 m^2/g and the surface of the sample was 3.5 cm^2, the mass of the 0.5 monolayer deposited on the sample was 0.3 µg. This estimation was used to determine the amount of surfactant nebulized in other experiments as well.

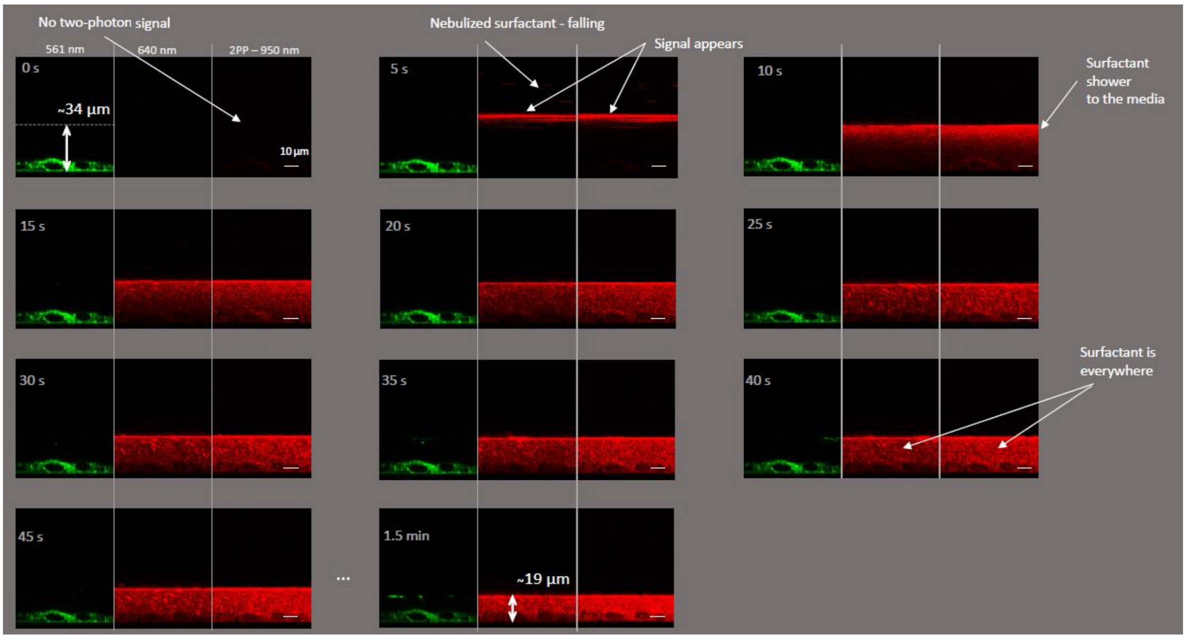

Figure S2: Time series of nebulizing Curosurf-StarRed, fluorescence microscopy at 561 nm, 640, and 950 nm. Most of the material is nebulized in the first 30 s, and the system settles in a few minutes;

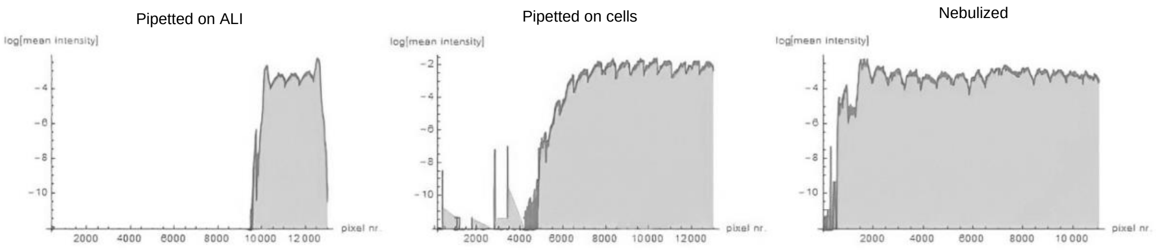

Figure S3: Intensity distribution of Curosurf-StarRed fluorescence, under different conditions
